# Career-Success Scale – A new instrument to assess young physicians' academic career steps

**DOI:** 10.1186/1472-6963-8-120

**Published:** 2008-06-02

**Authors:** Barbara Buddeberg-Fischer, Martina Stamm, Claus Buddeberg, Richard Klaghofer

**Affiliations:** 1 Department of Psychosocial Medicine, Zurich University Hospital, Zurich, Switzerland

## Abstract

**Background:**

Within the framework of a prospective cohort study of Swiss medical school graduates, a Career-Success Scale (CSS) was constructed in a sample of young physicians choosing different career paths in medicine. Furthermore the influence of personality factors, the participants' personal situation, and career related factors on their career success was investigated.

**Methods:**

406 residents were assessed in terms of career aspired to, and their career progress. The Career-Success Scale, consisting of 7 items, was developed and validated, addressing objective criteria of academic career advancement. The influence of gender and career aspiration was investigated by a two-factorial analysis of variance, the relationships between personality factors, personal situation, career related factors and the Career-Success Scale by a multivariate linear regression analysis.

**Results:**

The unidimensional Career-Success Scale has an internal consistency of 0.76. It is significantly correlated at the bivariate level with gender, instrumentality, and all career related factors, particularly with academic career and received mentoring. In multiple regression, only gender, academic career, surgery as chosen specialty, and received mentoring are significant predictors. The highest values were observed in participants aspiring to an academic career, followed by those pursuing a hospital career and those wanting to run a private practice. Independent of the career aspired to, female residents have lower scores than their male colleagues.

**Conclusion:**

The Career-Success Scale proved to be a short, reliable and valid instrument to measure career achievements. As mentoring is an independent predictor of career success, mentoring programs could be an important instrument to specifically enhance careers of female physicians in academia.

## Background

Depending on the career aspired to, medical school graduates plan their postgraduate training differently. In Switzerland, as in other German-speaking countries, nearly half of all young physicians aspire to work as a medical specialist in a private practice [1]. Another forty per cent pursue a hospital career, but only about ten percent aspire to an academic career. As found in our study and reported by other authors [2-4]* women physicians were generally less interested in academic pursuits than men*. At the beginning of the specialty training a greater number of residents think of an academic career but loose interest in it later on [5]. Main reasons assessed are lack of mentorship and role models, absence of faculty development programs, financial issues and difficulties balancing work and family [6,7]. Independent of the career aspired to, *performance should be assessed at regular intervals *to provide the trainees career adequate support [8]. It has been recognized that faculties need formalized career development strategies to improve the progress of their trainees [9,10]. *Mentorship *has been identified to be an important component of personal development, career guidance, career choice, and career success [11,12]. As experienced in a mentoring program [13], a continuous follow up of young physicians' career advancement and a feed-back of their performance motivates them, especially women, not to abandon their initial career aspiration. Blickle et al. [14] postulated that not only the quality of a mentor-protégé relationship but the sum of different supporting relationships, forming a kind of supporting network, reveals to be career relevant. Beside the institutional career support, a career-oriented *professional attitude and personality traits *such as instrumentality are positively associated with objective indicators of career success [15,16]. Furthermore, the *personal situation *often has an influence on career aspirations and success, in the sense of parents being role models (being academics), of receiving support by a partner, and of the amount of chores obligations; the latter two factors are more important for women [17]. As far as we know, there is no instrument to date measuring academic career performance in young physicians by different and objective criteria.

In the present paper *the term "Career" *is used in the narrow sense of a career to a prestigious position in the professional field of medicine, mainly in hospital medicine and/or in academics. This definition of "career" corresponds to the common meaning of career in German speaking countries.

In Switzerland, as in the other German speaking countries, physicians who aspire to a chief position in hospital or in academia have to be successful researchers, also those who are mainly clinicians. They have to fulfill the requirements for a habilitation (tenure track) (that means: at least 20 papers published as original papers in peer-reviewed English language journals with high impact factors in the field, being first author in at least half of the numbers of papers, also some book chapters and papers in other journals).

At the Zurich medical school, the only one in Switzerland, very experienced clinicians who have been medical educators for at least 5 years, can apply for the title of a "Klinischer Dozent" ("Clinical Lecturer"). Beside the teaching requirements they have to publish at least some papers addressing issues of clinical relevance. Usually medical educators are senior physicians in clinical medicine or in basic sciences who have already passed the tenure track (Habilitation); thus at Swiss medical schools, medical educators are researchers and advanced in their academic career.

Within the framework of a prospective survey of a cohort of Swiss medical school graduates [18-20], a Career-Success Scale (CSS) was developed in a sample of young physicians choosing different kinds of careers in medicine. The CSS aims to assess career achievements in an objective way. It measures academic career performance in a broader and more differentiated way than looking at the single aspects of research grants and publications. It was hypothesized (1) that male physicians are more advanced in their career than female physicians and score higher on the CSS, (2) that those aspiring to an academic career have higher values than those aspiring to a clinical career or to run a private practice. And it was hypothesized (3) that career success is significantly associated with personality factors, personal situation and career-related factors such as career motivation, specialty choice and mentoring experience.

## Methods

### Study design, sample development and study sample

The development of the Career-Success Scale is part of an ongoing *prospective survey of a cohort of graduates *of the three medical schools in German speaking Switzerland [18-20], beginning in 2001 (T1). Subjects were re-evaluated every two years. At the fourth assessment (T4) in 2007, the participants had worked as residents for five to six years; i.e. they have the same seniority at this stage of their career, similar positions and salary. To validate the CSS and to test the hypotheses, 406 (196 males, 48.3%; 210 females, 51.7%) residents aspiring to a career in private practice, hospital medicine or in academic medicine were included in the analyses. The mean age of the participants was 33.2 years (SD 2.22 ys, range 29 – 47 years). Forty-seven (24.5%) male and 36 (17.1%) female residents have children (p = 0.04).

To ensure participants' anonymity, the returned questionnaires were only identified by a code. The respondents sent their addresses to an independent address-administration office, allowing for follow-up. The study protocol was approved by the ethical committee of Zurich university.

### Instruments

All instruments are self-assessment scales. In the following, the main characteristics of the instruments and the constructs measured by them are described:

- *Questions concerning socio-demographic data *and *personal situation *(parents' education, partnership, household chore obligations)

- *Career aspired to *(private practice, hospital or academic career) and *chosen medical specialty*

- *Sense of Coherence Scale (SOC-13) *[21] (seven-point Likert scale), is a measure of a person's resistance to stress and his/her ability to manage stress.

- *Personal Attributes Questionnaire*, *GE-PAQ*, German Extended Personal Attributes Questionnaire [22], is a self-rating instrument for the assessment of gender-role orientation. Only the *Instrumentality (PAQ-I) *scale (8 items, six-point Likert scale) was used which contains instrumental traits (e.g. 'independent', 'decisive') that are considered to be socially desirable to some degree in both sexes but stereotypically more characteristic of males.

- *Career-Motivation Questionnaire *[23] (seven-point rating scale), only the *Extrinsic Career Motivation Scale *(i.e. striving for promotion, income, prestige) and the *Extraprofessional Concerns Scale *(i.e. prioritizing family, convenient working hours, job security) were applied.

- *Mentor-Protégé Relationships Questionnaire *[14] consists of five scales (Likert scale 0 – 4) measuring career-support type. We only used the *Networking scale *(4 items) and the *Support in career planning *scale (3 items). These two scales describe the crucial aspects of mentoring. Our data analyses show that the two scales are highly correlated (r = 0.71). Therefore we combined them to one scale named "*Mentoring-Experience Scale (MES)*", Cronbach's alpha = 0.92.

#### Career-Success Scale (CSS)

Based on results of previous assessments of the prospective Swiss cohort study, an expert meeting (clinicians and academics in their function as mentors and mentees, residency program directors, medical sociologist; equal gender distribution in the expert group) was held to define essential criteria for career success at this stage of physicians' postgraduate training. The experts agreed that research is the most relevant factor for pursuing a prestigious career in medicine (hospital and/or academia). Therefore they formulated seven items concerning scientific activities (i.e. number of research projects, lectures, publications, grants, criteria which correspond to the requirements for tenure track) (Table 1; see also Additional file 1). The introducing question of the CSS is: "Looking at your career, what career steps have you made until now?" Meaning and importance of the items are as follows:

**Table 1:** Items of the Career-Success Scale (CSS), the item values, factor loadings, communalities and the item-total correlations

**Item**	**Content**	**Item value**	**Factor loading**	**Communality**	**Item-total correlation r_it_**
1	Lectures/Talks at conferences	none = 0	0.65	0.43	0.57
		1 to 3 = 1			
		4 to 50 = 2			
2	Publications	None = 0	0.71	0.50	0.61
		1 = 1			
		2 to 3 = 2			
		4 and more = 3			
3	Collaboration in research project	no = 0, yes = 1	0.75	0.56	0.63
4	Months of research as principal activity	no = 0	0.75	0.56	0.54
		up to 9 months = 1			
		10 and more months = 2			
5	Scholarship awarded	no = 0, yes = 1	0.74	0.55	0.57
6	Competitively awarded third-party funds	no = 0, yes = 1	0.76	0.58	0.58
7	Research awards	no = 0, yes = 1	0.56	0.31	0.42

	**Career-Success Scale (CSS)**	**range of sum score = 0 – 11**	**Cronbach's alpha**	**0.76**	

*- Item 1 and 2: *Talks at conferences and publications are the core of an academic career, and thus a measure of professional productivity.

*- Item 3 and 4: *To pursue an academic career successfully, it is necessary to spend time in research at an early stage of one's career.

*- Item 5 – 7: *Achieving funding and awards provide an indication of quality and originality of the research.

### Statistical analyses

All analyses were carried out with SPSS for windows, release 15.0. To explore the dimensionality of the Career-Success Scale (CSS) we conducted a factor analysis (principal component analysis and variamax rotation); criteria for the number of factors were the number of eigenvalues ≥ 1 and the scree plot. Futhermore, the item-total-correlations and the reliability of the CSS (Cronbach's alpha) were computed. To investigate the influence of gender and career aspiration on career success we conducted a two-factorial analysis of variance (independent variables: gender and career aspiration, dependent variable: CSS). A multiple linear regression analysis was carried out to investigate the relationships between personality factors, personal situation, career related factors, and the CSS.

## Results

### Career aspiration and gender

In their sixth year of residency, the study participants responded as to what kind of career they aspire in the future. In Table 2 the distribution of participants according to gender, and career aspired to is listed. Significantly more females intend to work in a private practice than males, and significantly more males aspire to an academic career than females (p < .001). A hospital career is an equal option for doctors of either gender.

**Table 2:** Frequency distribution of participants according to gender and career aspired to

**Career aspiration**	**Males **n (%)	**Females **n (%)	**Total **n (%)
**Private practice**	79 (19.5)	113 (27.8)	192 (47.3)
**Hospital career**	85 (20.9)	87 (21.4)	172 (42.4)
**Academic career**	32 (7.9)	10 (2.5)	42 (10.3)

**Total**	196 (48.3)	210 (51.7)	406 (100)

Chi-square = 17.11, df = 2, p < 0.001

### Career-Success Scale (CSS)

**Figure 1:**
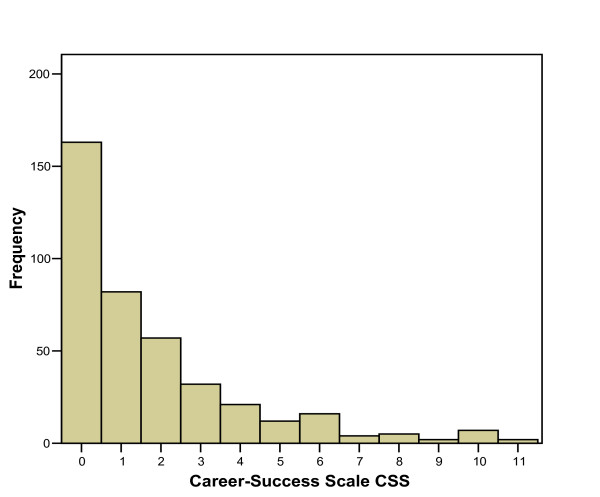
Distribution of the Career-Success Scale (CSS) (n = 406).

In Table 1 text and value of the items of the CSS are listed. The factor analysis of the 7 items yields one factor explaining 50% of variance. Also the factor loadings are shown, they range from 0.56 (item 7) to 0.76 (item 6), as well as the communalities, they range from 0.31 (item 7) to 0.58 (item 6). The sumscore of the CSS ranges from 0 to 11. The internal consistency (Cronbach's alpha) of the scale is 0.76. The distribution of the CSS in the study sample (n = 406) is seen in Figure 1; it is skewed towards the right, the mean value is 1.76 (SD = 2.33).

### Validation of the Career-Success Scale

It was analyzed whether the career success was different in participants aspiring to work in a private practice or intending to pursue a hospital or an academic career. As seen in Table 3, participants aspiring to an academic career have the highest CSS values, followed by those aspiring to a hospital career, the lowest indices are found in residents intending to work in a private practice. Female residents show lower Career-Success sumscores than male physicians in all types of career aspiration. There was no statistically significant interaction between gender and career aspiration.

**Table 3:** Means and standard deviations (SD) of Career-Success Scale (CSS) depending on gender and career aspired to; results of analysis of variance

	**Males **(n = 196)	**Females **(n = 210)	**Total **(n = 406)
**Career aspiration**	Mean (SD)	Mean (SD)	Mean (SD)
**Private practice **(n = 192)	1.18 (1.44)	0.61 (0.98)	0.84 (1.22)
**Hospital career **(n = 172)	2.19 (2.16)	1.25 (1.41)	1.72 (1.88)
**Academic career **(n = 42)	6.66 (2.28)	4.78 (2.49)	6.24 (2.80)

**Total **(n = 406)	2.51 (2.77)	1.05 (1.51)	1.76 (2.33)

**Effect**	**F**	**P**	**Partial eta squared**
Gender	F(1,400) = 21.77	0.001	0.05
Career aspiration	F(2,400) = 101.61	<0.001	0.34
Gender × Career aspiration	F(2,400) = 2.04	0.131	0.01

### Personality factors, personal situation, career related factors, and career success

As shown in Table 4, the CSS is significantly correlated at the bivariate level with gender, instrumentality, and all career related factors, particularly with academic career and received mentoring but not with the personal situation such as partnership and parents' education. Only gender, academic career, surgery as chosen specialty, and received mentoring have a significant beta weight on CSS in multiple regression. The adjusted R square is 0.50 (p < 0.001).

**Table 4:** Multiple regression for Career-Success Scale (CSS) as dependent variable (bivariate correlations and beta weights)

**Independent variables**	**Bivariate correlation with CSS**	**Beta weight in multiple regression**
**Personality factors**		
- Gender (female)	-0.32***	-0.12**
- Sense of Coherence	0.05	0.01
- Instrumentality	0.24***	0.04
**Personal situation**		
- Parents' education (academics)	0.02	-0.02
- Partnership (yes)	0.05	0.04
- Doing the chores myself	-0.06	0.06
**Career related factors**		
- Extrinsic career motivation	0.19***	0.06
- Extraprofessional concerns	-0.30***	-0.04
- Academic career (yes)	0.66***	0.56***
- Specialty choice (surgery)	0.31***	0.10*
- Mentoring	0.34***	0.15***

***Adjusted R Square***		0.50***

* p < 0.05, ** p < 0.01, *** p < 0.001

Furthermore we analysed whether having children has an influence on career success in female and male participants. The gender specific multiple regression analyses did not reveal a significant influence of children on career success.

## Discussion

### Career aspiration and gender

As reported in the literature [2-4,19,24], more male physicians aspire to an academic career, and are in the top ranks of faculty posts. Though there is a greater awareness for gender equality to date, gender equity in terms of fairness and justice for men and women in the professional opportunity structure is far from being realized. Whether female physicians themselves follow different professional and personal career goals than males, or whether they do not have equal access to academic career-relevant resources, cannot be distinguished with data of the present study.

### Development of the Career-Success Scale

Within the framework of a prospective Swiss cohort study, the present paper reports on the development of the CSS, validated in a sample of 406 young physicians in their sixth year of residency. Regular assessment of career progress is important to give trainees the support they need at the respective stage of their individual career [8,25]. If there are any, career obstacles can be identified early and helped to be overcome. In academic medicine, evaluation of career performance mainly focuses on research activities, grants, publications, and faculty posts. We developed a new Career Success Scale because there are no scales available to date, which assess relevant and objective career performance factors in young physicians.

### Validation of the Career-Success Scale

In terms of the content validity participants aspiring to an academic career have by far the highest CSS values. If this would not be the case, the validity of the scale would not be given in principle. The CSS fulfills essential test-statistical criteria: The scale proved to be unidimensional by factor analysis, has a sufficient internal consistency, and high item-total correlations respectively.

### Associations between personality factors, personal situation, career-related factors and career success

While at the bivariate level there were significant correlations with instrumentality and all career-related factors, in the multivariate analysis only specialty choice and mentoring remained significant predictors. As already found in a previous study [20], instrumentality, gender and surgery as specialty choice are correlated. It is known that one or another variable miss the significance of the prediction, depending on the respectively applied multivariate model. Instrumentality as a proactive personality trait is mentioned by other authors to be associated with both self-reported objective and subjective indicators of career success [15]. Mentoring as a crucial factor in career support and success is found in our study and reported in several other studies [11,26,27]. Contrary to reports in the literature [17], the personal situation, i.e. parents' education, partnership, chores obligations, and having children does not contribute to the prediction model in our study.

Some *limitations *of the present study have to be considered. The CSS only measures external factors relevant for an academic career. The scale does not measure a person's achievement of an individual career goal, i.e. it is not assessed whether a person has achieved what she/he aspired to.

## Conclusion

The CSS proved to be a short, reliable and valid instrument to measure career progress in residents by objective criteria. The new instrument is easy to administer, well accepted and should be tested in further studies, also in other countries. As mentioned by other authors [8] and experienced in an own mentoring program [13], objectively assessed evaluations of career progress should be established early in physicians' postgraduate training and at regular intervals to supply adequate and individual career support. Mentoring is an independent predictor of career success. Therefore mentoring programs might contribute to enhance the number of medical academics, especially females.

## Competing interests

The authors declare that they have no competing interests.

## Authors' contributions

BB–F is principal investigator, designed the study, participated in the development of the Career Success Scale (CSS), interpreted the data, and drafted the manuscript. RK participated in the study design and the development of the CSS, performed the statistical analyses, and critically revised the drafts of the paper. MS participated in the development of the CSS, and critically revised the drafts of the paper. CB participated in the study conception, contributed methodological advise, and critically revised the drafts of the paper.

## Pre-publication history

The pre-publication history for this paper can be accessed here:



## Supplementary Material

Additional file 1 Career-Success Scale English and German version. The wording of the 7 items of the Career-Success Scale are given in English and in German. Click here for file
