# Supplementary material for: Career-Success Scale – A new instrument to assess young physicians' academic career steps
Source: BMC Health Serv Res. 2008 Jun 2;8:120. doi: 10.1186/1472-6963-8-120 (PMC2442432; doi:10.1186/1472-6963-8-120)
Supplement: Additional file 1 — Career-Success Scale English and German version. The wording of the 7 items of the Career-Success Scale are given in English and in German. [file 1472-6963-8-120-S1.pdf]

Career-Success Scale (CSS) English version

Looking at your career, what career steps have you made until now?

1. Lectures and talks held at conferences and scientific meetings (without advanced training in your clinic)	No	❑0	
	Yes	❑ ⇨	How many?
2. Publications (original papers with peer review, first-authorship or co-authorship)	No	❑0	
	Yes	❑ ⇨	How many?
3. Collaboration in a larger research project	No	❑0	
	Yes	❑1	
4. Research as principal activity	No	❑0	
	Yes	❑ ⇨	How many moths in total?
5. Scholarship awarded	No	❑0	
	Yes	❑1	
6. Competitively gained third party funds for research	No	❑0	
	Yes	❑1	
7. Research awards	No	❑0	
	Ja	❑1	

Career-Success Scale (CSS) German version

Wenn Sie Ihre berufliche Laufbahn überblicken, welche Leistungen haben Sie bis jetzt erbracht?

1. Eigene Fachvorträge an Konferenzen, Kongressen, Tagungen (ohne Klinik-interne Fortbildungen) gehalten	Nein<br>Ja	❑0<br>❑ ⇨	 Wie viele?
2. Eigene Publikationen (Originalarbeiten mit peer-review, Erst- oder Mitautorenschaft) verfasst	Nein<br>Ja	❑0<br>❑ ⇨	 Wie viele?
3. Mitarbeit in einem grösseren Forschungsprojekt	Nein<br>Ja	❑0<br>❑1	
4. Forschung als Haupttätigkeit	Nein<br>Ja	❑0<br>❑ ⇨	 Wie viele Monate gesamthaft ?
5. Forschungsstipendium	Nein<br>Ja	❑0<br>❑1	
6. Drittmittel für Ihre Forschungstätigkeit eingeworben (Nationalfonds, Stiftungen, Industrie)	Nein<br>Ja	❑0<br>❑1	
7. Forschungsauszeichnung (z.B. Forschungspreise) erhalten	Nein<br>Ja	❑0<br>❑1	
